# Vergleich der radikalen Zystektomie mit der trimodalen Therapie beim muskelinvasiven Harnblasenkarzinom

**DOI:** 10.1007/s00066-023-02134-1

**Published:** 2023-08-02

**Authors:** M. Fleischmann, C. Rödel

**Affiliations:** 1 Klinik für Strahlentherapie und Onkologie, Universitätsklinikum Frankfurt, Goethe-Universität, Theodor-Stern-Kai 7, 60590 Frankfurt, Deutschland; 2 Deutsches Konsortium für Translationale Krebsforschung (DKTK), Partnerstandort Frankfurt, Frankfurt, Deutschland; 3 Frankfurt Cancer Institute (FCI), Frankfurt, Deutschland

## Hintergrund und Fragestellung

Die trimodale Therapie (TMT), bestehend aus initialer transurethraler Tumorresektion gefolgt von einer kombinierten Radiochemotherapie, ist bei Patientinnen und Patienten mit muskelinvasivem Harnblasenkarzinom eine primär organerhaltende Alternative zur radikalen Zystektomie. Für einen direkten Vergleich beider Therapieformen sollen die jeweiligen Tumorkontroll- und Überlebensraten mittels *„propensity score matching“ (PSM) *verglichen werden.

## Patienten und Methoden

Diese retrospektive PSM-Analyse umfasste 722 Patientinnen und Patienten mit einem cT2–4N0M0-Urothelkarzinom der Harnblase, die zwischen 2005 und 2017 an drei nordamerikanischen Universitätskliniken (Boston, Los Angeles, Toronto) behandelt wurden (440 mittels radikaler Zystektomie, 282 mittels TMT). Das Patientenkollektiv musste sich prinzipiell für beide Therapieformen eignen und einen solitären Tumor (< 7 cm) ohne extensives oder multifokales Carcinoma in situ mit allenfalls unilateraler Hydronephrose sowie eine prätherapeutisch adäquate Blasenfunktion aufweisen. Primärer Endpunkt der Studie war das metastasenfreie Überleben. Als sekundäre Endpunkte wurden das krankheitsspezifische, krankheitsfreie und Gesamtüberleben untersucht. Unterschiede wurden mithilfe des „propensity score matching“ (PSM) und der inversen Wahrscheinlichkeitsgewichtung (IPTW) analysiert.

## Ergebnisse

Die Analyse erfolgte in einer balancierten, 3:1 *gematchten* Kohorte. Das mediane Follow-up betrug 4,4 Jahre nach Zystektomie und 4,9 Jahre nach TMT. Bei austarierten, prätherapeutisch-klinischen Prognosefaktoren (bezüglich Alter, Geschlecht, ECOG-Status, BMI, cTN-Kriterien, Hydronephrose, Carcinoma in situ, Durchführung einer [neo-]adjuvanten Chemotherapie) war das metastasenfreie 5‑Jahres-Überleben in den beiden Therapiegruppen identisch (74 %). Das krankheitsspezifische 5‑Jahres-Überleben betrug 83 % nach Zystektomie und 85 % nach TMT, das Gesamtüberleben favorisiert mit 72 % gegenüber 77 % sogar die TMT (HR 0,75, *p* = 0,0078). Die Analysen waren auch mit der IPTW-Methode robust und unterschieden sich nicht nach Zentren und zusätzlich durchgeführten Sensitivitätsanalysen.

## Schlussfolgerung der Autoren

Diese multiinstitutionelle Studie liefert die bislang beste Evidenz, dass die onkologischen Ergebnisse nach radikaler Zystektomie und TMT in einem selektionierten, aber hinsichtlich Prognosefaktoren austarierten Patientenkollektiv vergleichbar sind. Daher sollte die TMT allen geeigneten Patientinnen und Patienten mit muskelinvasivem Harnblasenkarzinom als Alternative zur Zystektomie angeboten werden und nicht nur solchen, die wegen signifikanter Komorbiditäten nicht für eine radikale Operation infrage kommen.

## Kommentar

Randomisierte Studien für den Vergleich der radikalen Zystektomie mit der primär organerhaltenden TMT des muskelinvasiven Blasenkarzinoms mit Level-1-Evidenz zu Tumorkontroll- und Überlebensraten, funktionellen Endpunkten, Toxizität und Lebensqualität fehlen. Eine solche Studie (Selective bladder Preservation against Radical Excision [SPARE]) war in Großbritannien aufgelegt worden, wurde bei schlechter Rekrutierung jedoch vorzeitig abgebrochen [1].

Methodisch sind retrospektiv vergleichende und populationsbezogene Studien durch die Diskordanz zwischen klinischer und pathologischer Stadieneinteilung, unterschiedliche Selektionskriterien und eine ungleiche Verteilung von prognostischen Faktoren limitiert und liefern oft diskrepante Ergebnisse. So ergab z. B. eine Metaanalyse mit insgesamt 12.380 Patienten (8330 nach Zystektomie, 4050 nach TMT) keine signifikanten Unterschiede im krankheitsspezifischen 10-Jahres-Überleben (HR 0,83, *p* = 0,39) und Gesamtüberleben (HR 1,02, *p* = 0,9), während eine populationsbezogene Studie der National Cancer Data Base der USA mit 12.843 behandelten Patientinnen und Patienten (11.586 nach Zystektomie, 1257 nach TNT) ein schlechteres Langzeitüberleben der primär organerhaltend therapierten Patienten beschrieb [2, 3]. Daher stellen *Propensity-score-*Analysen, wie die hier vorliegende, die gegenwärtig bestmögliche Methodik und Evidenz für einen Vergleich dar.

Die onkologischen Ergebnisse beider Therapiestrategien sind exzellent und belegen die hohe Behandlungsqualität an den 3 Standorten: So betrug die mediane Anzahl an untersuchten pelvinen Lymphknoten bei Zystektomie 39, die R1-Resektions-Rate lag bei nur 1 % und die Lokalrezidivrate bei 3 %. Auch in solch erfahrenen Zentren erreicht die perioperative Mortalität der radikalen Zystektomie allerdings eine Größenordnung von 2,5 %, was zu der verminderten Gesamtüberlebensrate im Vergleich zur TMT (die keine therapieassoziierte Mortalität aufwies) beigetragen haben mag. Nach TMT zeigten 11 % einen muskelinvasiven, 20 % einen nichtmuskelinvasiven Rest- oder Rezidivtumor. Eine Salvage-Zystektomie wurde in 13 % der Fälle durchgeführt. Das krankheitsfreie Überleben nach Salvage-Zystektomie war äquivalent zu dem der nicht zystektomierten Patientinnen und Patienten und unterstreicht deren kuratives Potenzial.

Zu beachten ist, dass es sich hier um ein selektioniertes Patientenkollektiv handelte mit solitären cT2–4N0M0-Tumoren ohne extensives oder multifokales Carcinoma in situ, allenfalls unilateraler Hydronephrose sowie mit adäquater Blasenfunktion. Interessanterweise wiesen nur 30 % aller an den Kliniken im Beobachtungszeitraum zystektomierten Patientinnen und Patienten die oben genannten Kriterien auf, die sie auch für die TMT qualifiziert hätten. Dies unterstreicht erneut die Wichtigkeit einer adäquaten Selektion der Patientinnen und Patienten für die TMT. Andererseits musste sich das hier untersuchte TMT-Kollektiv prinzipiell auch für eine Zystektomie eignen, um eine Imbalance bezüglich Alter, Komorbiditäten, ECOG etc. zu vermeiden.

Bei vergleichbaren Tumorkontroll- und Überlebensraten wären Angaben zu funktionellen Endpunkten und der Lebensqualität des hier untersuchten Patientenkollektivs von großer Bedeutung gewesen, konnten aber offensichtlich wegen der retrospektiven Natur der Analyse nicht valide erhoben werden. Langzeitdaten aus den größten monoinstitutionellen Serien sowie den multizentrischen, prospektiven RTOG-Studien zu TMT berichten über chronische Grad-3- bis Grad-4-Toxizitäten in einer Größenordnung von 5 bis 6 % für urogenitale sowie 2 bis 3 % für gastrointestinale Nebenwirkungen [4]. Daten zur Lebensqualität vor und bis zu 5 Jahre nach TMT, die auf Grundlage des *Functional Assessment of Cancer Therapy-Bladder* (FACT-BL) im Rahmen der prospektiven BC2001-Studie erhoben wurden, zeigen, dass sich die Blasenfunktion zum Ende der TMT zwar signifikant verschlechterte, nach 6 Monaten aber schon wieder die Ausgangswerte erreichte [5].

Neue Entwicklungen zur weiteren Optimierung der TMT betreffen derzeit die Präzisionsradiotherapie durch die bildgeführte, adaptive Strahlentherapie [6] sowie die Integration von Immuntherapeutika. Die INTACT-1806-Studie sowie die KEYNOTE-992-Studie testen gerade die TMT mit oder ohne Atezolizumab bzw. Pembrolizumab in einem Phase-III-Design.

### Fazit

In diesem nach Prognosekriterien balancierten *Propensity-score-*Vergleich sind die onkologischen Endpunkte der TMT mindestens vergleichbar zur radikalen Zystektomie, wenn nicht besser. Diese Daten begründen erneut die auch in der deutschen S3-Leitlinie hinterlegte Empfehlung, dass die „primär“ organerhaltende, multimodale Therapie Patienten mit lokal begrenztem, muskelinvasivem Urothelkarzinom (cT2–4 cN0/Nx M0) angeboten werden sollte, die sich nicht für eine radikale Zystektomie eignen *oder die eine Alternative zur radikalen Operation anstreben* [7].

*Maximilian Fleischmann und Claus Rödel, Frankfurt*
